# Removal of Zearalenone from Degummed Corn Oil by Hydrolase on a Batch-Refining Unit

**DOI:** 10.3390/foods11233795

**Published:** 2022-11-24

**Authors:** Chenwei Zhao, Pengkai Xie, Jun Jin, Qingzhe Jin, Xingguo Wang

**Affiliations:** State Key Laboratory of Food Science and Technology, International Joint Research Laboratory for Lipid Nutrition and Safety, School of Food Science and Technology, Jiangnan University, 1800 Lihu Road, Wuxi 214122, China

**Keywords:** zearalenone, hydrolase, degummed corn oil, degradation rate, response surface experiment

## Abstract

The removal of zearalenone (ZEN) from degummed corn oil (DCO) using hydrolase on a batch-refining unit was studied. According to single-factor and response surface experiments, the optimum technological conditions for reaching the maximum degradation rate were a temperature of 39.01 °C, a pH of 8.08, a time of 3.9 h, and an enzyme dosage of 44.7 mg/kg, whereby the rate of ZEN degradation can reach 94.66%. Different effects on the removal of ZEN were observed at different initial ZEN contents under the optimal technological conditions, of which the decrease was rapid for high ZEN content and slow for low ZEN content.

## 1. Introduction

Zearalenone (ZEN) is a non-steroidal estrogen mycotoxin, which is mainly produced by *Fusarium roseum*, *Fusarium graminearum*, *Fusarium tricinum*, and other *Fusarium* microorganisms during their secondary metabolism [1]. ZEN has attracted much attention because of its damage and toxicity to the reproductive system, immune system, liver, kidney, and genetic material of humans [2,3,4,5,6]. ZEN is mainly found in moldy corn, wheat, barley, and other cereals, as well as their by-products. It is the most widely contaminated mycotoxin in the world [7]. Corn is easily polluted by ZEN in the process of planting and harvesting [8]. As ZEN is a benzoic acid lactone compound and has a certain lipophilicity, it is very easy to be transferred into crude corn oil during oil extraction. The highest content of ZEN in corn crude oil was more than 8000 μg/kg in some reports [9]. The European Union (EU) standard (ec1126-2007) stipulated the ZEN limit in refined corn oil, where it should be limited within 400μg/kg [10]. Thus, ZEN in crude corn oil needs to be removed. According to the physical and chemical properties of ZEN, its removal methods mainly include physical detoxification, chemical degradation, and biological degradation.

The chemical degradation method of ZEN for corn oil focuses on alkali refining. The lactone bond of ZEN could be hydrolyzed by alkali under alkaline conditions to produce ZEN-Na, which is dissolved in water and removed from crude oil. The removal rate can reach more than 44% [11]. Adsorption and deodorization are the physical processes that can remove ZEN, in which the toxin is adsorbed by adsorbents such as activated clay and activated carbon, reaching a 5.57–17.35% removal rate [12]. ZEN is substantially removed from corn crude oil by deodorization under the action of steam stripping at high temperatures and vacuums. It is reported that, when the deodorization temperature rose from 210 to 270 °C, the removal rate increased from 36.38% to 99.00% [13]. However, the higher the temperature, the easier it is to produce *trans* fatty acids (TFA) [14].

Compared with the other two methods, biological degradation is relatively mild. It is not easy to cause the loss of food nutrients, and it has the characteristics of strong specificity, high conversion rate, and little environmental pollution [15]. It has become the research hotspot of ZEN degradation and removal. Biodegradation refers to an adsorption method using microorganisms. The degradation of intracellular and extracellular microorganisms, including live bacteria detoxification and enzyme degradation, degrades ZEN into low toxic or non-toxic products [16].

Bacteria degradation is the suggested technique to reduce the toxicity of ZEN to animals [17]. At present, the microorganisms used for the biodegradation of ZEN are mainly bacteria, fungi, and molds, specifically including *Bacillus*, *Lactobacillus*, *yeast*, and *Rhizopus*. Ping et al. [18] studied and obtained *Bacillus licheniformis* CK1, which can degrade 98% ZEN in corn meal culture for 36 h. Bakutis et al. [19] isolated four yeasts with ZEN clearance function, namely *Rhodotorula rubra*, *Rhodotorula glutinis*, and *Geotrichum fermentans*, with clearance rates that were 100%, 93.2%, 45%, and 44% respectively. In 2010, Vekiru et al. [20] studied the mechanism of degradation of ZEN by *Trichosporon mycotoxin* and found that the lactone ring of ZEN was hydrolyzed in that the product had no toxicity by in vitro test.

Most of the above-reported strains can degrade ZEN in the culture medium, and there is no report on their application in corn oil. Many scientists also extract and enrich enzymes with good decomposition ability from the intracellular, extracellular, and other fungi or plants of microorganisms to degrade ZEN. In 2002, Takahashi Ando et al. [21] obtained an alkaline hydrolase ZHD101 from *Clonostachys Rosea* IFO 7063 for the first time. After expression by *Schizosaccharomyces pombe* and *Escherichia coli*, it was found that the enzyme can hydrolyze ZEN. Subsequently, Takahashi Ando [22,23] found E Coli and sZhd101 that was expressed in *Saccharomyces cerevisiae*, exhibiting different ZEN degradation abilities. Popiel et al. [14] isolated three enzymes from *Clonostachys Rosea*, namely AN169, AN154, and AN171. These three enzymes can degrade 2 mg/mL ZEN after a 4 H reaction, with degradation rates of 20%, 25%, and 98%, respectively.

However, there are few reports on the enzymatic removal of ZEN from corn oil. Only Chang et al. [24] reported that adding ZLHY-6 enzyme to alkali refined corn crude oil at the enzyme activity of 1.5 mg/mL, a reaction temperature of 40–45 °C, and time of 2–5 h, reducing the ZEN content from 617.45 to 13.00 µg/kg, with a removal rate of 97.89%.

In this paper, through single factor and response surface experiments, the optimal reaction conditions in the degradation of corn crude oil ZEN were determined, and the degradation effects of the optimal process on corn oil ZEN with different ZEN contents were investigated.

## 2. Materials and Methods

### 2.1. Materials

Degummed corn oil (DCO) was obtained from different corn oil factories in China with ZEN contents of 994, 2967, 5089, and 7066 μg/kg, respectively. For ZEN hydrolase obtained from Rongvezyme preparation Co., Ltd. in Zhuhai, China, the enzyme activity was 243 U/g, as measured according to the methods reported by Wang [25]. ZEN standard was purchased from Sigma-Aldrich (St. Louis, MO, USA) along with Tris, hydrochloric acid, and other solvents and chemicals, which were of analytical grade.

### 2.2. Enzymatic Degradation Experiment

DCO (500 g) and 0.1 mol/L Tris-HCI buffer (3 mL) solutions of different pH and a certain dosage of ZEN hydrolase were added to a batch-reactor (Shenke, Wuxi, China). The sample was then added to a high-speed shear machine (IKA ULTRA—TURRAX UTL2000, IKA Co., Ltd., Staufen, Germany) to mix the samples at 4900 g for 2 min. The mixed sample (100 g) was then placed into an enzyme reactor at a specified temperature. After a certain time, the temperature was increased to 95 °C to terminate the reaction. The sample was then separated using centrifugation at 9800× *g* for 10 min. The upper oil layer was dried at 80 °C with 0.09 mPa.

### 2.3. Detection of ZEN Content

The concentrations of ZEN in the corn oil before and after processing were determined by a high-performance liquid chromatography with a fluorescence detector (Waters 2695, Waters Co., Ltd., Milford, MA, USA) in accordance with GB 5009.209-2016 (National Standard of the People’s Republic China, 2016).

Separation was conducted by a C18 column (4 µm particle size, 150 × 4.6 mm, Waters Co., Ltd., Milford, MA, USA) at 25 °C, and the injection volume was set as 100 µL. A mobile phase consisting of acetonitrile-water-methanol [46:46:8, *v*/*v*/*v*] was used at a flow rate of 1 mL/min.

### 2.4. Statistical Analyses

All experiments were independently carried out in triplicate, and SPSS (version 19.0, SPSS, Inc., Chicago, IL, USA) was used for the data analysis. Results are presented as mean ± standard deviation of triplicate measurements.

## 3. Results and Discussion

### 3.1. Effects of Reaction Temperature on Enzymatic Degradation of ZEN in DCO

**Figure 1:**
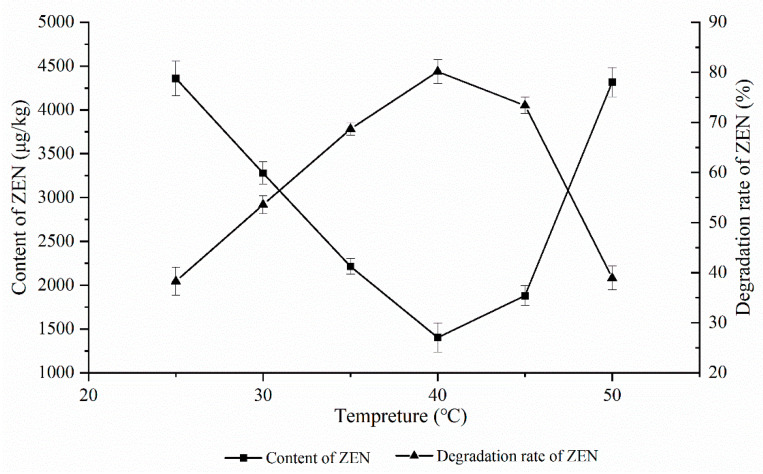
Effects of reaction temperature on the degradation of zearalenone.

As shown in Figure 1, the effect of the ZEN degrading enzyme on the degradation of ZEN in corn degumming oil was investigated at 25, 30, 35, 40, 45, and 50 °C under a reaction time of 2 h, a pH of 7.5, and an enzyme dosage of 20 g/kg.

An enzyme is a type of active protein whose catalytic activity is greatly affected by temperature. Under the optimal temperature, the maximum catalytic activity of an enzyme is achieved. It is reported that the optimum reaction temperature of the ZEN degrading enzyme is 35–40 °C.

It can be seen from Figure 1 that, for a gradient decrease ranging from 25 to 40 °C, the peak and valley were reached at 40 °C, followed by a rise. When the temperature increased from 45 to 50 °C, the ZEN content rose sharply. On the one hand, the enzyme activity gradually increased with the increasing temperatures. As the temperature improved, the oil viscosity decreased, and the probability of oil–water two-phase contact increased. When the temperature was excessively high, the ZEN degrading enzyme was rapidly inactivated, resulting in a low degradation rate of only 37.2% when the temperature was 50 °C. Therefore, 40 °C was chosen as the optimum reaction temperature.

### 3.2. Effects of pH Value on Enzymatic Degradation of ZEN in DCO

**Figure 2:**
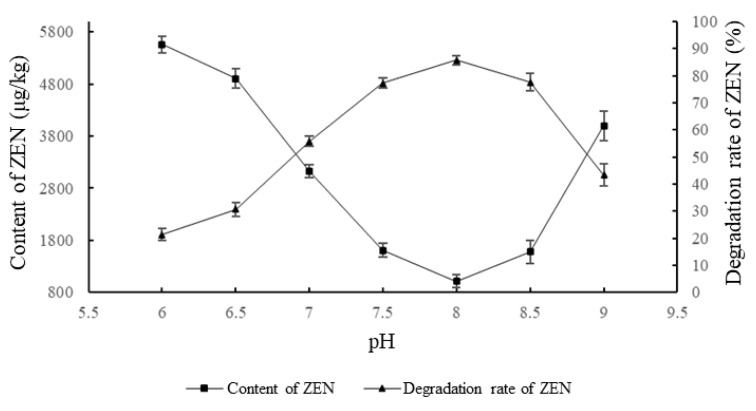
Effects of reaction pH on the degradation of zearalenone.

As shown in Figure 2, the effect of the ZEN degrading enzyme on ZEN degradation in corn degumming oil at different pH values was investigated when the reaction time was 2 h, the amount of the enzyme was 30 g/kg, and the reaction temperature was 40 °C.

pH is an important factor that affects the catalytic activity of an enzyme [26]. At a certain pH, an enzyme will exhibit maximum activity. Beyond the tolerance of the enzyme itself, the structure of the enzyme changes, and the activity decreases or is even lost. It can be seen from Figure 2 that the ZEN content in the corn oil first decreased and then increased with the increase in pH value, while the trend for the degradation rate was the opposite. Under acidic conditions, the enzyme activity was low. When the pH was neutral, the degradation rate reached 55.76%. When the pH was in a weak alkaline state, the enzyme activity was high. The highest degradation was observed when the pH was 8. As the pH continued to rise after this point, the activity decreased. When the pH was 9, the degradation rate was only 46%. This is because, under weakly alkaline conditions, the enzyme hydrolyzes the lactone bond of ZEN to produce hydrolyzed ZEN. The product is readily soluble in light alkaline solution, thus leaving the oil–water reaction interface and causing the hydrolysis reaction to proceed in the direction of hydrolysis. When the pH is excessively high, the active group of the enzyme is destroyed, leading to decreased activity. Therefore, a pH of 8 was chosen as the optimum reaction pH.

### 3.3. Effects of Reaction Time on the Enzymatic Degradation of ZEN in DCO

**Figure 3:**
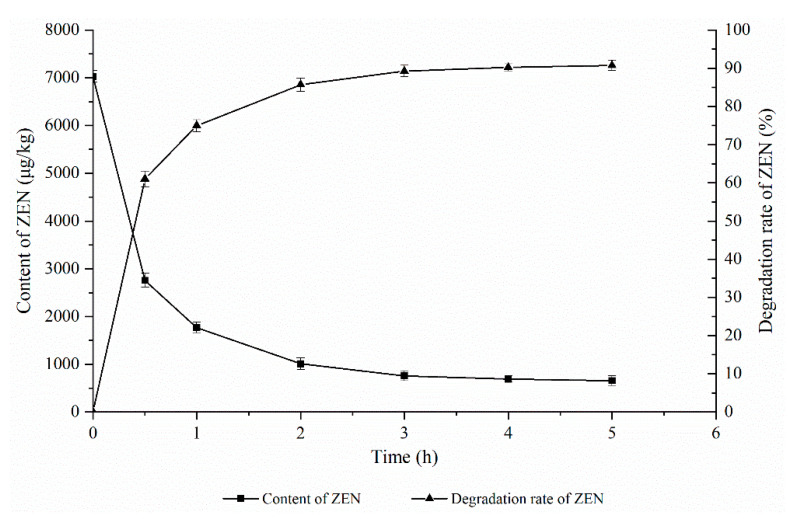
Effects of reaction time on the degradation of zearalenone.

As shown in Figure 3, the effect of the ZEN degrading enzyme on the degradation of ZEN in corn degumming oil was investigated at a reaction temperature of 40 °C, an enzyme dosage of 30 g/kg, and a pH of 8.

Reaction time is an important factor in the enzymatic degradation process. If the reaction time is too short, the enzymatic hydrolysis reaction will not be completed, the ZEN content will be too high, and the product accumulation will not reach the maximum value, resulting in a waste of resources. If the reaction time is too long, however, there will be an increase in energy consumption and production costs, which is not conducive to industrial production. It can be seen from Figure 3 that the ZEN content in corn oil decreased rapidly with the extension of time and then stabilized. When the reaction time reached 3 h, the ZEN content in the corn oil was basically stable, which may be due to reaching the equilibrium of the ZEN hydrolysis reaction in this state. Therefore, 3 h was chosen as the optimal reaction time.

### 3.4. Effects of Enzyme Dosage on the Enzymatic Degradation of ZEN in DCO

**Figure 4:**
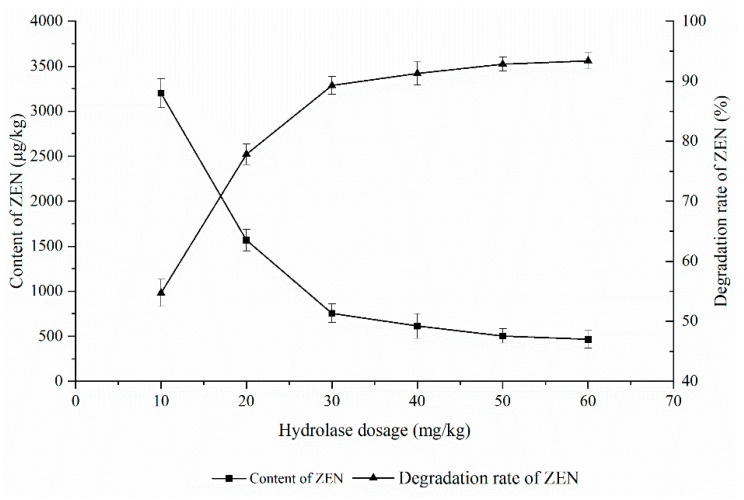
Effects of enzyme dosage on the degradation of zearalenone.

As shown in Figure 4, the effect of the ZEN degrading enzyme on the degradation of ZEN in corn degumming oil was investigated at a reaction temperature of 40 °C, a pH of 8, and a reaction time of 3 h.

The hydrolase dosage affects the reaction speed. The more hydrolase, the greater the chance of contact with the substrate and the faster the reaction speed. However, when the hydrolase dosage is excessive, it is close to saturation relative to the substrate, and the impact is small. It can be seen from Figure 4 that the ZEN content in the corn oil decreased rapidly with an increasing enzyme addition before becoming stable. When 10 mg/kg of the enzyme was added, the degradation rate of ZEN was only 54%. With the increase in the enzyme, the content of ZEN decreased rapidly. When the added amount exceeded 40 mg/kg, there was little change in ZEN content. This is because the amount of effective enzyme that can react remained unchanged despite the relatively higher increase of enzyme; in other words, there is a so-called “enzyme saturation” phenomenon [27]. According to the research in Section 2, when the amount of enzyme added is 40 mg/kg, the content of ZEN in degraded corn oil can reach 755.7 μg/kg, which meets the requirements of deodorization. Therefore, 40 mg/kg was selected as the optimal enzyme amount.

### 3.5. Optimization of the Enzymatic Degradation of ZEN in DCO Using Response Surface Methodology

Many factors have been found to affect the enzymatic degradation of ZEN in corn oil. The degrading enzyme is a hydrolase, and its reaction speed is related to the relative concentrations of substrate and water. However, since the enzyme is a water-soluble enzyme, the molar ratio of water to ZEN is far more than the theoretical amount of 1:1 under the current addition amount, and water is in excess; thus, the molar ratio of the substrate was not considered.

Response surface analysis experiments with four factors and three levels were designed using the Design-Expert software. The response surface analysis results are presented in Table 1.

Taking temperature (A), pH (B), time (C), and hydrolase dosage (D) as independent variables, the average ZEN degradation rate obtained through the experiment was taken as the response value. The experimental data in Table 1 were regressed using Design-Expert software. The three-factor quadratic multiple regression model for the ZEN content was obtained as follows:

ZEN degradation rate (%) = 86.95 + 1.79A + 0.91B + 4.05C + 11.84D + 2.32AB − 3.20AC − 3.19AD − 0.14BC − 0.91BD − 1.33CD − 6.71A^2^ − 3.32B^2^ + 0.34C^2^ − 3.57D^2^. Response surface software was used to verify the model reliability. The variance analysis of the regression model is shown in Table 2.

It can be seen from Table 2 that the *p* value of the model was less than 0.0001, indicating that the model equation was extremely significant, and the mismatch term F = 4.55, *p* = 0.0787 > 0.05, indicating that the term was not significant. This shows that the test data were not significantly correlated with the model, the model was reliable, and the accuracy and reliability of the test were high. The difference between the model correction determination coefficient Adj R-Squared = 0.8853 and the model prediction coefficient Pred R-Squared = 0.6890 was less than 0.2, which indicates that the response value of the model was high, and the Adeq precision was 16.4656 > 4, which indicates that the model can effectively reflect the test accuracy. Therefore, this model can be used to analyze and predict the degradation rate of ZEN. In addition, the influence of each influencing factor on the experiment is d > c > a > b; that is, the amount of enzyme added > time > temperature > time, and the amount and time of the enzyme added had a significant effect on the experimental results.

**Figure 5:**
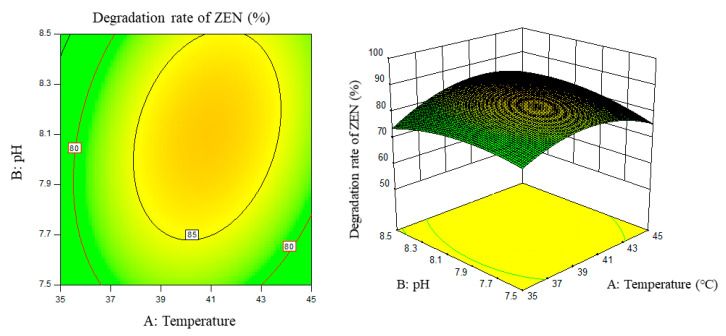
Temperature, pH, and their interaction vs. the response surface and contour line of the degradation rate of ZEN.

**Figure 6:**
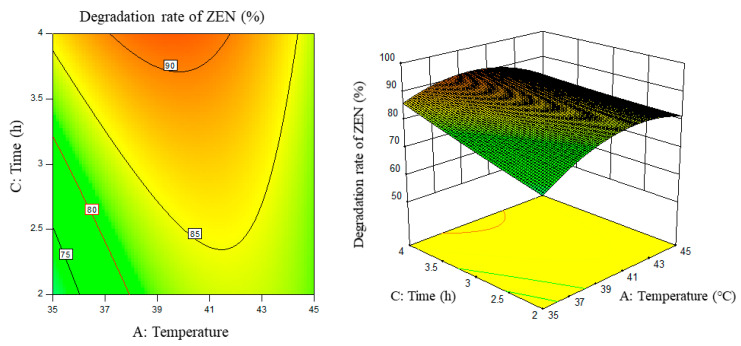
Temperature, time, and their interaction vs. the response surface and contour line of the degradation rate of ZEN.

**Figure 7:**
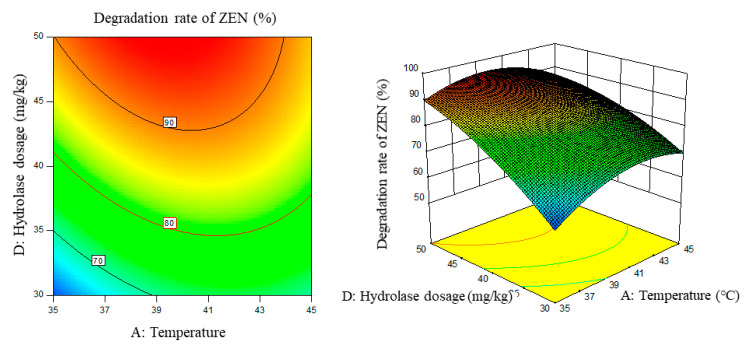
Temperature, enzyme dosage, and their interaction vs. the response surface and contour line of the degradation rate of ZEN.

**Figure 8:**
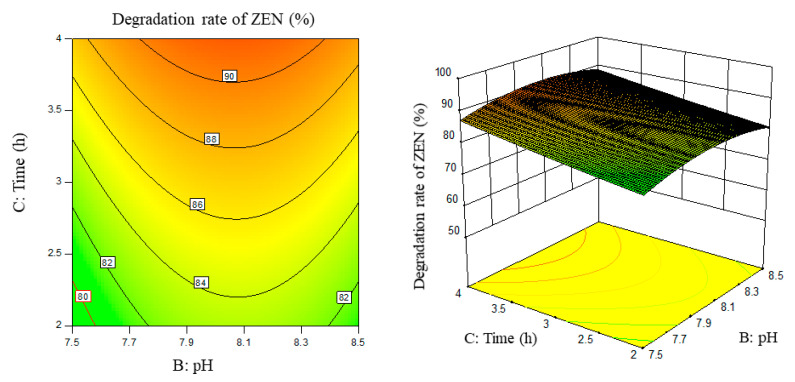
PH, time, and their interaction vs. the response surface and contour line of the degradation rate of ZEN.

**Figure 9:**
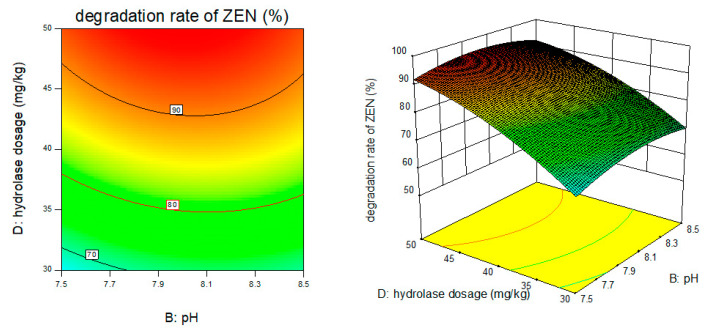
PH, enzyme dosage, and their interaction vs. the response surface and contour line of the degradation rate of ZEN.

**Figure 10:**
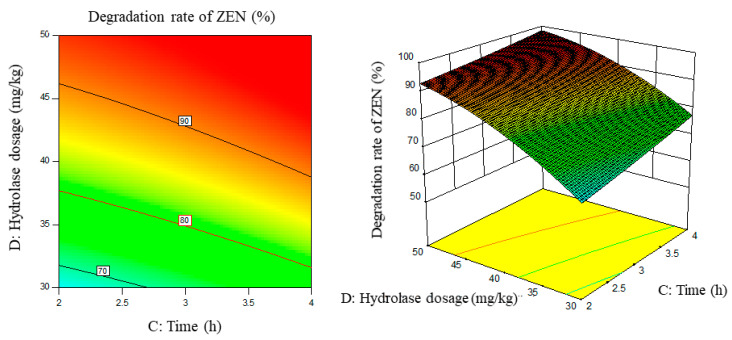
Time, enzyme dosage, and their interaction vs. the response surface and contour line of the degradation rate of ZEN.

Response surface results are shown in Figure 5, Figure 6, Figure 7, Figure 8, Figure 9 and Figure 10.

From the above six figures, it can be seen that the response value of the ZEN degradation rate first increased and then decreased with the increase in temperature and pH, in addition to increasing with the increase in time and excess enzyme ratio, which is consistent with the results of the single-factor test.

The following procedure was used to determine the optimal process conditions. In order to reach the maximum ZEN degradation rate, Design-Expert was used to process the equation within the selected conditions. It was found that there were 86 solutions to the equation. When the temperature was 39.01 °C, the pH was 8.08, the time was 3.9 h, and the enzyme dosage was 44.7 mg/kg, the ZEN degradation rate could reach 95.37%. Three parallel experiments were carried out under these conditions. The actual degradation rate measured by the experiment was 94.66%, which is basically consistent with the predicted results and, moreover, verified the reliability of the response model.

### 3.6. Effects of the Optimum Technological Conditions on the Degradation of ZEN in DCO with Different Initial ZEN Contents

**Figure 11:**
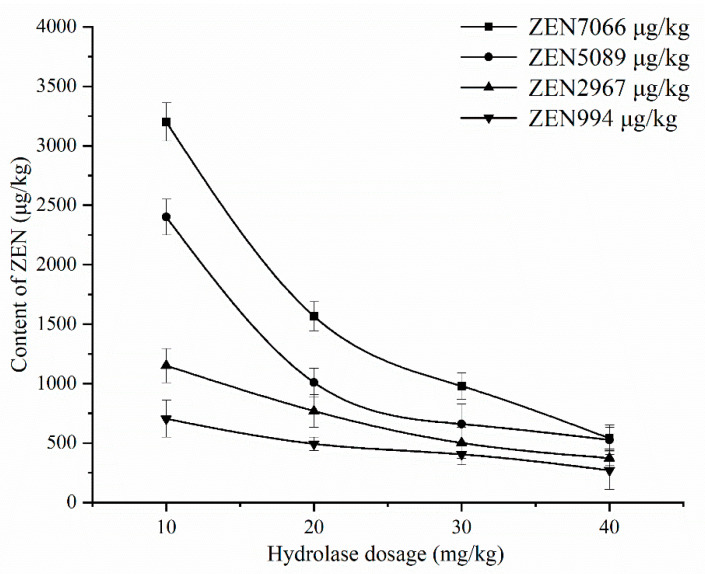
The effect of hydrolase dosage on the degradation of ZEN with different initial contents of ZEN in DCO.

The optimal process obtained from the above response surface experiment was used for corn degumming oil with a high ZEN content (7066 μg/kg). When the ZEN content in corn oil decreased, the substrate concentration of enzyme reaction decreased, and the applicability of the above best process still needs to be investigated. It was found from the above research that the hydrolase dosage had the greatest impact on the degradation rate of ZEN. Therefore, as shown in Figure 11, DCO with different initial contents of ZEN were selected to investigate the effect of different dosages of ZEN hydrolase on the degradation rate of ZEN under the same conditions of other processes.

It can be seen from Figure 11 that the content of ZEN in corn oil with different initial ZEN content decreased with the increase in hydrolase dosage. When the ZEN content was high (initial ZEN contents of 7066 and 5089 μg/kg), the ZEN content sharply decreased, whereas when the ZEN content was low (initial ZEN content of 2967 and 994 μg/kg), the ZEN content decreased slowly. This is because when the ZEN content is high, the substrate concentration during the enzymatic degradation is high, and the reaction speed is fast. ZEN is thus rapidly hydrolyzed. When the ZEN content is low, the substrate concentration is low, the reaction speed is relatively slow, and the ZEN content decreases slowly. Therefore, for corn oil with different initial ZEN contents, the hydrolase dosage should be determined according to ZEN content to minimize the cost to the greatest extent.

## 4. Conclusions

Using DCO as raw material, the optimal reaction conditions of the ZEN degrading enzyme were determined through single-factor and response surface experiments, and the adaptability of the conditions to the detoxification effect of corn oil was investigated for different ZEN contents. The following conclusions were obtained:

(1) According to the single-factor and response surface experiments, the optimum technological conditions for reaching the maximum degradation rate are a temperature of 39.01 °C, a pH of 8.08, a time of 3.9 h, and an enzyme dosage of 44.7 mg/kg, where the ZEN degradation rate can reach 94.66%.
(2) Through the enzymatic hydrolysis of DCO with different initial ZEN contents under the optimal technological conditions, different trends were observed for different ZEN contents. At high ZEN content, ZEN is rapidly hydrolyzed, whereas the ZEN content decreases slowly the initial content is low.

## Figures and Tables

**Table 1:** Box-Behnken experimental design matrix and experimental results.

Item	A	B	C	D	Content of ZEN (µg/kg)	Degradation Rate of ZEN (%)
	Temperature (°C)	pH	Time/h	Hydrolase Dosage/mg/kg		
1	40	8.5	4	40	765.29	89.17
2	40	8.5	3	30	1964.31	72.20
3	35	8	4	40	864.36	87.77
4	35	7.5	3	40	1689.34	76.09
5	40	8	3	40	966.63	86.32
6	40	8.5	2	40	1451.33	79.46
7	40	8	3	40	903.74	87.21
8	40	8	2	30	2365.98	66.52
9	35	8.5	3	40	1689.78	76.09
10	45	8.5	3	40	1489.25	78.92
11	35	8	3	30	3085.02	56.34
12	40	8	2	50	531.26	92.48
13	40	7.5	4	40	730.91	89.65
14	40	7.5	2	40	1456.71	79.39
15	35	8	2	40	1978.65	72.00
16	40	8	3	40	1107.95	84.32
17	35	8	3	50	756.32	89.30
18	40	8	4	30	1826.32	74.15
19	40	7.5	3	30	2166.44	69.34
20	40	8.5	3	50	599.36	91.52
21	45	8	2	40	1246.82	82.35
22	45	8	4	40	1037.29	85.32
23	40	7.5	3	50	543.38	92.31
24	45	8	3	50	599.32	91.52
25	40	8	4	50	368.32	94.79
26	45	8	3	30	2026.53	71.32
27	40	8	3	40	806.96	88.58
28	40	8	3	40	825.31	88.32
29	45	7.5	3	40	2143.28	69.67

**Table 2:** Analysis of variance for each term in the fitted regression model.

Source	Sum of Squares	df	Mean Square	F-Value	*p*-Value	Significance
Model	2422.08	14	173.01	16.43	<0.0001	**
A	38.61	1	38.61	3.67	0.0762	
B	9.89	1	9.89	0.9392	0.3489	
C	197.26	1	197.26	18.73	0.0007	**
D	1681.31	1	1681.31	159.68	<0.0001	**
AB	21.45	1	21.45	2.04	0.1754	
AC	40.99	1	40.99	3.89	0.0686	
AD	40.69	1	40.69	3.86	0.0695	
BC	0.0775	1	0.0775	0.0074	0.9329	
BD	3.34	1	3.34	0.3168	0.5824	
CD	7.11	1	7.11	0.6749	0.4251	
A²	291.99	1	291.99	27.73	0.0001	**
B²	71.37	1	71.37	6.78	0.0208	**
C²	0.7412	1	0.7412	0.0704	0.7946	
D²	82.77	1	82.77	7.86	0.0141	**
residual	147.41	14	10.53			
Lack of fit	135.50	10	13.55	4.55	0.0787	
Pure error	11.91	4	2.98			
Cor total	2569.49	28				
R-Squared = 0.9426, Adj R-Squared = 0.8853, Pred R-Squared = 0.6890, Adeq Precisior = 16.4656						

Note: ** is extremely significant, i.e., *p* < 0.01; A, temperature; B, pH; C, time; D, hydrolase dosage.

## Data Availability

The data used to support the findings of this study can be made available by the corresponding author upon request.
